# Associations Between the Readiness for Return to Work Scale and Return to Work: A Prospective Study

**DOI:** 10.1007/s10926-017-9705-2

**Published:** 2017-03-16

**Authors:** Lene Aasdahl, Kristine Pape, Chris Jensen, Ottar Vasseljen, Tore Braathen, Roar Johnsen, Marius Steiro Fimland

**Affiliations:** 1 Department of Public Health and General Practice, Faculty of Medicine, NTNU, Norwegian University of Science and Technology, Postboks 8905 MTFS, 7491 Trondheim, Norway; 2 National Center for Occupational Rehabilitation, Rauland, Norway; 3 University College of Southeast Norway, Porsgrunn, Norway; 4 Hysnes Rehabilitation Center, St. Olavs Hospital, Trondheim University Hospital, Trondheim, Norway

**Keywords:** Rehabilitation, Sick leave, Scale, Mental health, Musculoskeletal diseases

## Abstract

*Purpose* To explore the usefulness of the Readiness for return to work scale in individuals participating in occupational rehabilitation, by assessing the association between the scale and return to work (RTW), and comparing the scale to a question assessing individuals’ expectations about length of sick leave. *Method* Prospective cohort study with 9 months follow-up. Participants took part in one of two randomized clinical trials. Associations between the Readiness for RTW scale and RTW was analyzed using linear and logistic regression, with adjustment for age, gender and education. The Readiness for RTW scale was compared to a self-reported question assessing participants’ expectations about length of sick leave using adjusted/pseudo R^2^. *Results* For participants not working (n = 96), high scores on two dimensions (Prepared for action—self-evaluative and Prepared for action—behavioral) were associated with a higher probability of sustainable RTW and more working days. For those working (n = 121), high scores on the Uncertain maintenance dimension was associated with a lower probability of sustainable RTW and less working days. Generally, models including the Readiness for RTW dimensions were not as good at explaining work outcomes as models including a single expectation question. Stage allocation, allocating participants to the dimension with the highest score, was problematic due to several tied scores between (not necessarily adjacent) dimensions. *Conclusions* Three of the Readiness for RTW dimensions were associated with RTW. However, several weaknesses with the Readiness for RTW scale were established and we particularly do not recommend the stage allocation approach for clinical use in its current form.

**Electronic supplementary material:**

The online version of this article (doi:10.1007/s10926-017-9705-2) contains supplementary material, which is available to authorized users.

## Introduction

Return to work (RTW) after long-term sick leave is a complex and dynamic process described in several conceptual models [1–3]. One such model is the Readiness for RTW model [4], which is based on the stages of change model [5] and the phase model of occupational disability [6]. The Readiness for RTW model suggests that RTW is a process where the person on sick leave progresses through different dimensions or stages of change towards RTW. These stages are precontemplation (not intending to RTW), contemplation (considering RTW), preparation (making plans to RTW), action (RTW), and maintenance (staying at work). Three dimensions of change have been suggested to mediate the progression through the stages: self-efficacy, the individual’s decisional balance and change processes concerning RTW [4]. Change processes can be both mental (thoughts, feelings and attitudes) and behavioral. During the first stages, thoughts and feelings are gradually oriented towards the need for change, then in the later stages actual change in behavior manifests, like contacting the employer [4].

The RTW-process is dependent on the dynamic interaction between a person’s health status and contextual factors [3]. Hence, it is important to have instruments which can capture the dynamic nature of the RTW process [3]. However, currently no such instruments have solid empirical support. Based on the Readiness for RTW model, Franche et al. [7] developed and psychometrically validated the Readiness for RTW scale in a Canadian cohort. They identified four underlying factors for individuals not working and two for individuals working, corresponding to the stages in the Readiness for RTW model (Table 1). More recently; however, a validation study in persons referred to occupational rehabilitation in Norway found fewer factors; two for people not working, and two for people working [8]. Two of these four factors were later found to be associated with future work participation in the only published longitudinal investigation of the Readiness for RTW scale [9].

**Table 1:** Description of the different stages in the Readiness for RTW scale

Stage	Description
Individuals not working	Individuals who are 100% sick listed
Precontemplation	The person is not thinking about starting behavior change with regards to RTW
Contemplation	The person has started to think about returning to work, but is still ambivalent and has no concrete plans
Prepared for action—self-evaluative	The person seeks information about RTW and make concrete plans for RTW
Prepared for action—behavioral	The RTW plans are set into action
Individuals working	Individuals who are partly or fully working (including graded sick leave)
Uncertain maintenance	The person has returned to work, but is struggling to stay at work
Proactive maintenance	The person has found good strategies for staying at work

Franche et al. [7] suggested that the Readiness for RTW scale could be used for evaluation of interventions and also clinically in tailoring stage-specific interventions. This is tested in an ongoing Danish study where the individual’s rehabilitation program is tailored based on the allocated Readiness for RTW stage [10]. The Readiness for RTW model is considered promising as it captures the dynamics of the RTW process [3]. However, limited research has been performed on the Readiness for RTW model and the Readiness for RTW scale [7–9], and more research is needed on this instrument before it can be applied clinically [3, 11].

In two randomized clinical trials evaluating different occupational rehabilitation programs [12, 13], all participants were asked to answer the Readiness for RTW scale before and after the rehabilitation program. In the current study we assessed the association between the Readiness for RTW scale dimensions and future RTW in persons with musculoskeletal or mental health disorders participating in occupational rehabilitation. We also assessed whether a model including the Readiness for RTW dimensions or a model including a self-reported question assessing the participants` expectations about length of sick leave best described work outcomes, as a single expectation question has been associated with RTW in previous studies [14–17].

## Methods

### Study Design and Participants

We conducted a prospective cohort study with 9 months follow-up in individuals participating in one of two randomized clinical trials including three different rehabilitation programs. The purpose of the randomized trials were to assess the effect on sickness absence of two different inpatient multicomponent occupational rehabilitation programs versus a less comprehensive outpatient program. More details about the randomized trials have been published in a protocol article [12], and in a study assessing effects on health outcomes [13]. The study was approved by the Regional Committee for Medical and Health Research Ethics in Central Norway (No.: 2012/1241), and the trial is registered in clinicaltrials.gov (No.: NCT01926574).

Eligible participants were 18–60 years of age, sick listed 2–12 months with a diagnosis within the musculoskeletal (L), psychological (P) or general and unspecified (A) chapters of the ICPC-2 (International Classification of Primary Care, Second edition). The current sick leave status at inclusion had to be at least 50% off work. Exclusion criteria were: (1) alcohol or drug abuse; (2) serious somatic (e.g., cancer, unstable heart disease) or psychological disorders (e.g., high suicidal risk, psychosis, ongoing manic episode); (3) specific disorders requiring specialized treatment; (4) pregnancy; (5) currently participating in another treatment or rehabilitation program; (6) insufficient oral or written Norwegian language skills to participate in group sessions and fill out questionnaires; (7) scheduled for surgery within the next 6 months; or (8) serious problems with functioning in a group settings.

### The Rehabilitation Programs

*The inpatient programs* consisted of group-based Acceptance and Commitment therapy (ACT) [18], individual and group-based physical training, mindfulness and individual meetings with the coordinators in work-related problem-solving sessions and creating a RTW plan. One program lasted 3.5 weeks and the other 4 + 4 days (with 2 weeks at home in-between). Both programs lasted 6–7 h each day. The programs took place at Hysnes rehabilitation center, established as part of St. Olavs Hospital, in central Norway. *The outpatient program* consisted mainly of group-based ACT. The sessions were held at the Department of Physical Medicine and Rehabilitation at St. Olavs Hospital once a week for 6 weeks, each session lasting 2.5 h. A more detailed description of the programs has been published elsewhere [12].

### Questionnaires

Self-reported data on the Readiness for RTW scale and other questionnaires were collected via internet-based questionnaires at the start and end of the rehabilitation programs.

The Readiness for RTW scale [7] consists of two parts, part A is answered by individuals who are 100% sick listed and part B is answered by individuals who are working (includes graded sick leave). Part A consists of 22 items and part B of 12 items (Online Resource 1). Each item is answered on a 5-point scale from “strongly disagree” to “strongly agree”. The wording of two questions was changed from “pain” and “injury” in the original scale to “health complaints” in the Norwegian version to include participants with other complaints. In the study by Franche et al. the items reflected four dimensions (hereafter referred to as the original dimensions) for individuals not working: precontemplation (items A1, A4, A22); Contemplation (A15, A20, A21); Prepared for action—self-evaluative [A9, A12R (reversed item scale); A13, A18] ​(A9, A12R (reversed item scale), A13, A18); and Prepared for action—behavioral (A6, A10, A11). For individuals working there were two dimensions; Uncertain maintenance (B8, B9, B10, B11R, B12) and Proactive maintenance (B2, B5, B6, B7). Franche et al. [7] described two approaches for scoring the questionnaire: (1) the multidimensional approach recommended for research; and (2) the stage allocation approach recommended for clinical use. In the multidimensional approach, a mean score is calculated for the items it comprises (range 1–5), whereas in the stage allocation approach, the individual is allocated to the one stage where they have the highest mean score. When using the multidimensional approach Franche et al. [7] used the term “dimension” for the different stages, while for the stage allocation approach they used the term “stage”. The dimensions found in the Norwegian validation study [8] (hereafter referred to as the Braathen dimensions) were RTW inability (A1, A4, A10R, A22) and RTW uncertainty (A18, A20, A21) for individuals not working, corresponding to the precontemplation and contemplation dimensions in the original scale. For individuals working they found uncertain work maintenance (B2R, B6R, B8, B10) and proactive work maintenance (B5, B7, B12R).

Expectations about length of sick leave were recorded using the question “For how long do you believe you will be sick listed from today?” with six response options “not at all”, “less than 1 month”, “1–2 months”, “2–4 months”, “4–10 months” and “more than 10 months”. Categories “not at all”, “less than 1 month” and “1–2 months” were combined to one category “less than 2 months” in the analyses, as these categories were close in time and included few participants.

Data on possible confounders such as age, gender, anxiety and depression symptoms (measured using The Hospital Anxiety and depression scale (HADS) [19]), length of sick leave, education and job status (having employment or not) were recorded at baseline.

### Sick Leave Register Data

Sick leave was measured using data from the National Social Security System Registry, where all individuals receiving any form of benefits in Norway are registered by their social security number. Medically certified sick leave is compensated with 100% coverage for the first 12 months. The first 16 days are covered by the employer, the rest by the Norwegian Welfare and Labour Administration. After 12 months of sick leave more long-term benefits may be offered in the form of work assessment allowance and disability pension, which both covers approximately 66% of the income. Individuals on work assessment allowance are supposed to participate in modified work, but if this is not possible for medical reasons, the individual and the case manager develop a plan for later work resumption.

The data consisted of registrations of benefits from four different sources: sick-leave payments, sick leave certificates, work assessment allowance and disability pension. Participants were followed for 9 months after they ended the rehabilitation programs.

Two different measures of RTW were constructed: (1) *Sustainable RTW* was defined as 1 month without receiving medical benefits during follow-up and (2) *Work participation days* was measured as number of days not receiving medical benefits during follow-up, adjusted for graded sick leave, employment fraction and calculated as a 5-day work week.

### Statistical Analysis

The main analyses were based on the original dimensions by Franche et al. [7], and separate analyses were conducted according to work status (working/not working) at the end of rehabilitation. We used linear and logistic regression to assess associations between scores on the Readiness for RTW scale dimensions (four dimensions for those not working and two for those working) and the two RTW measures. The main analyses were adjusted for age, gender and education. Education level was dichotomized as high (college/university) or low. We used the results from the adjusted regression analyses to estimate the predicted probability of sustainable RTW and work participation days using average adjusted predictions (i.e., predictions were made with covariates constant at their means).

As the linearity assumption was not fully satisfied the analyses were repeated with the dimension scores categorized into 1.0–1.9, 2.0–2.9, 3.0–3.9 and 4.0–5.0. The analyses were also performed using the stage allocation approach described above. Changes in the dimension scores from before to after rehabilitation were compared using the Wilcoxon signed rank test, as they were not normally distributed. Associations between the single expectation question and RTW were assessed by logistic and linear regression, as in the main analyses.

We compared how the Readiness for RTW dimensions and the single expectation question explained work outcomes using adjusted R^2^/pseudo R^2^. First we compared models including the different dimensions, both as continuous and categorical variables, but also with interactions between the dimensions. Secondly we compared models including the different dimensions with a model including the single expectation question.

We performed the following sensitivity analyses: (1) adjustment for type of rehabilitation program in addition to age, gender and education, (2) adjustment for length of sick leave at inclusion, total HADS score and whether the participant had a job (for those not working) in addition to the variables in the main analyses, and (3) we repeated the main analyses without participants who failed to answer the questionnaire within 30 days after rehabilitation. Cronbach’s alpha coefficients were calculated for both the original and the Braathen dimensions to assess the average covariance between items in the associated dimension construct.

We considered p-values (two-tailed) <0.05 to be statistically significant. Precision was assessed using 95% confidence intervals. All analyses were done using STATA 14.1 (StataCorp. 2015. Stata Statistical Software: Release 14. College Station, TX: StataCorp LP).

## Results

In total, 217 participants (65%) answered the Readiness for RTW scale questionnaire at the end of the rehabilitation program and were included in this study (of the 334 participants who participated in the randomized clinical trials). Of these, 96 participants filled out the “not working” part of the questionnaire and 121 filled out the “working” part. Table 2 shows baseline characteristics of the two groups. Of the participants not working, 28 (29%) achieved sustainable RTW during 9 months follow-up and the median number of work participation days was 80 (interquartile range (IQR) 27–139). Of those working, 78 (64%) participants achieved sustainable RTW and the median number of work participation days was 165 (IQR 111–189).

**Table 2:** Participants’ characteristics at the end of the rehabilitation programs (baseline in the main analyses)

	Not working (n = 96)^a^	Working (n = 121)^b^
Age mean (SD)^c^	47 (9.6)	47 (8.5)
Women n (%)	76 (79%)	102 (84%)
Higher education n (%)^c,d^	37 (39%)	58 (48%)
Employment fraction before inclusion n (%)^c^		
No work	20 (21%)	1 (1%)
Full time	51 (53%)	86 (71%)
Part time	17 (18%)	26 (21%)
Graded disability pension	8 (8%)	8 (7%)
HADS mean (SD)		
Anxiety (0–21)	7.7 (4.4)	7.4 (3.9)
Depression (0–21)	6.1 (4.1)	6.0 (4.1)
Pain level mean (SD)		
Average pain (0–10)	4.1 (2.1)	4.1 (2.1)
Expectations about length of sick leave n %		
<2 months	24 (25%)	31 (26%)
2–4 months	29 (30%)	29 (24%)
4–10 months	21 (22%)	22 (18%)
>10 months	11 (11%)	14 (12%)
missing	11 (11%)	25 (21%)
Main diagnosis for sick-leave (ICPC-2) n (%)^e^		
A—general and unspecified	8 (8%)	10 (8%)
L—musculoskeletal	48 (50%)	70 (58%)
P—psychological	40 (42%)	41 (34%)
Length of sick leave at inclusion^e,f^		
Median days (IQR)	232 (176–285)	215 (180–266)
Readiness for Return to work		
Median (IQR)	1.0 (1.0-1.7)	
Precontemplation (1–5)	4.0 (3.3–4.3)	
Contemplation (1–5)	2.5 (2.0–3.5)	
Prepared for action- self-evaluative (1–5)	4.0 (3.3–4.7)	
Prepared for action- behavioral (1–5)		
Uncertain maintenance (1–5)		3.4 (2.6–4.0)
Proactive maintenance (1–5)		4.3 (3.9–4.5)

^a^100% sick leave

^b^Graded sick leave/working

^c^Measured at inclusion in the randomized trials

^d^Higher (tertiary) education: college or university

^e^Based on data from the National Social Security System Registry

^f^Number of days on sick leave during the last 12 months prior to inclusion. Measured as calendar days, not adjusted for graded sick- leave

Cronbach’s alphas for the original dimensions were: Precontemplation 0.78, contemplation 0.72, Prepared for action-self-evaluative 0.64, Prepared for action-behavioral 0.59, Uncertain maintenance 0.65 and Proactive maintenance 0.70. Cronbach’s alphas for the Braathen dimensions were: RTW inability 0.64, RTW uncertainty 0.66, Uncertain work maintenance 0.41 and Proactive work maintenance 0.55. The original dimensions were therefore used in the subsequent analyses.

### Associations Between the Readiness for RTW Scale and Work Outcomes: Multidimensional Approach

**Fig. 1:**
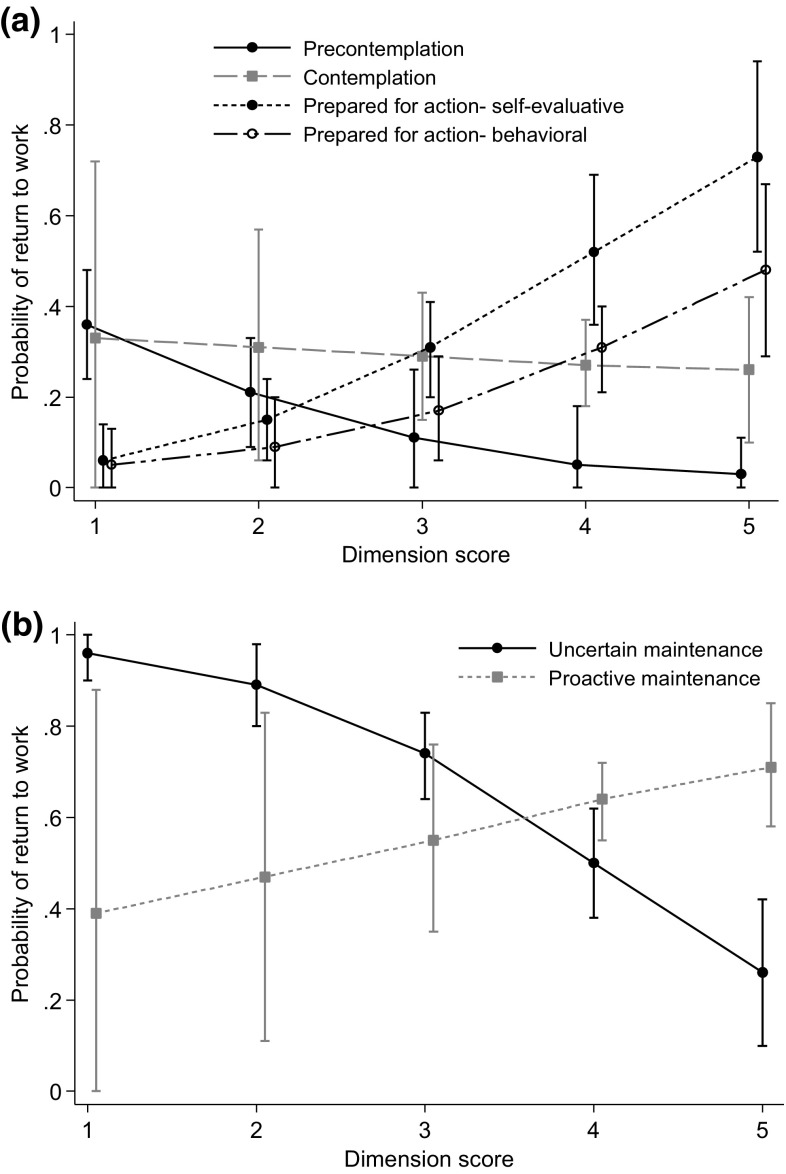
Estimated probabilities (with 95% confidence intervals) for sustainable return to work during 9 months of follow-up for the different dimensions (scale scores 1–5) in the **a** not working sample and **b** working sample. Analyses performed with logistic regression, adjustment for age, gender and education. For both samples N varied somewhat according to the number of missing information on each variable. Dimension scores measured at the end of rehabilitation

**Fig. 2:**
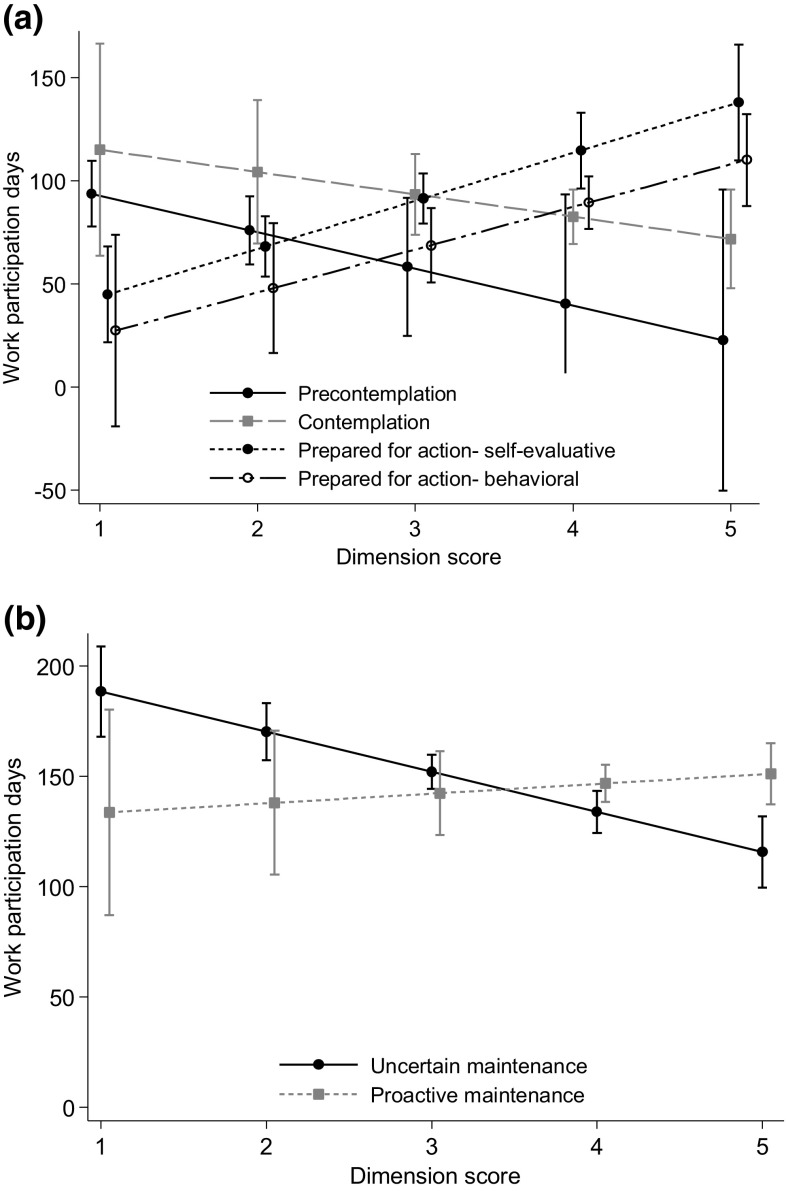
Estimated number of work participation days (with 95% confidence intervals) during 9 months follow-up for the different dimensions (scale 1–5) in the **a** not working sample and **b** working sample. Analyses performed with linear regression, adjustment for age, gender and education. For both samples N varied somewhat according to the number of missing information on each variable. Dimension scores measured at the end of rehabilitation

For individuals not working two of the four dimensions were associated with a higher probability of sustainable RTW and more work participation days (Figs. 1, 2 and Online Resource 2); Prepared for action—self-evaluative (p < 0.001) and Prepared for action—behavioral (p = 0.01–0.02). For persons working, high scores on the uncertain maintenance dimension was associated with a lower probability of sustainable RTW (p < 0.001) and fewer work participation days (p < 0.001). None of the sensitivity analyses changed the conclusions. However, the associations were less clear when the main analyses were performed with the dimensions as categorical variables, but for the Prepared for action—self-evaluative and Uncertain maintenance dimensions some of the categories were still statistically significant (results not shown).

### Associations Between the Readiness for RTW Scale and Work Outcomes: Stage Allocation Approach

When using the stage allocation approach in the not working group 16 participants obtained the same score on two dimensions, in which 12 had the same score on two dimensions that were not adjacent regarding progression towards work (Contemplation and Prepared for action—behavioral). One participant had equal scores on three dimensions. Excluding participants with ties gave two participants with the highest score in the Precontemplation stage, 40 in Contemplation, 4 in Prepared for action—self-evaluative, 31 in Prepared for action—behavioral, 91 in Uncertain maintenance and 25 in Proactive maintenance. When excluding the Precontemplation and Prepared for action—self-evaluative stages due to low numbers of persons, those in the prepared for action-behavioral stage had a higher probability of sustainable RTW than those in the contemplation stage and more work participation days in the follow-up period (Online Resource 3). Persons in the Uncertain maintenance stage had a higher probability for sustainable RTW and more work participation days than those in the Proactive maintenance stage.

### Changes During Rehabilitation

Comparing scores at the start and the end of the rehabilitation programs showed a statistically significant increase in scores for three of the dimensions: Prepared for action—self-evaluative, Prepared for action—behavioral and Proactive maintenance (Online Resource 4). The change scores for Uncertain maintenance (p = 0.08) and Prepared for action—self-evaluative (p = 0.05) tended to be associated with work participation days during follow-up, and Uncertain maintenance also for sustainable RTW (p = 0.07). However, none of the change scores were statistically significantly associated with sustainable RTW or work participation days during follow-up (results not shown).

### The Readiness for RTW Scale Versus a Single Expectation Question

The single question assessing the participants’ expectations about length of sick leave was associated with both sustainable RTW and work participation days (Table 3). For those working, there was a graded association between expected length of sick leave and work participation. For those not working there was not a clear association, but a noticeable difference between the less than 2 months category and the others. Although small, there was a larger explained variance in models including the single expectation question compared to models including the different Readiness for RTW dimensions (Online Resource 5). Combining the different Readiness for RTW dimensions in the same model (according to work status), somewhat increased the explained variance, but still not more than the single expectation question.

**Table 3:** Associations between a single question assessing participants’ expectations about length of sick leave answered at the end of rehabilitation and work outcomes during 9 months follow-up

	Estimated work participation days (95% CI)^a^	Probability of sustainable return to work (95% CI)^b^
Expectations about length of sick leave for participants not working (n = 85)		
<2 months	127 (104–150)	0.65 (0.47–0.84)
2–4 months	72 (51–94)	0.14 (0.01–0.27)
4–10 months	61 (36–86)	0.18 (0.02–0.34)
>10 months	62 (28–97)	0.08 (0.00–0.22)
Expectation about length of sick leave for participants working (n = 96)		
<2 months	167 (153–181)	0.87 (0.75–0.99)
2–4 months	138 (123–152)	0.57 (0.39–0.74)
4–10 months	122 (105–139)	0.39 (0.19–0.59)
>10 months	96 (74–117)	0.15 (0.00–0.33)

^a^Estimated from linear regression analyses with adjustment for gender, age and education (set at their mean)

^b^Estimated from logistic regression analyses with adjustment for gender, age and education (set at their mean)

## Discussion

Three of the Readiness for RTW dimensions were associated with work outcomes. For participants not working, high scores on the Prepared for action—self-evaluative and Prepared for action—behavioral scale were associated with a higher probability of sustainable RTW and more work participation days. For those working, high scores on Uncertain maintenance was associated with a lower probability of sustainable RTW and less work participation days. Allocating participants to the dimension with the highest score was problematic due to several tied scores between (not necessarily adjacent) dimensions. Models including the Readiness for RTW dimensions were generally not as good at explaining work outcomes as models including a single expectation question.

The association between the Readiness for RTW scale and work outcomes has only been investigated in one previous study [9]. In that study Braathen and co-workers, based on fewer dimensions, reported an association between two of the dimensions and RTW: RTW inability (corresponds to precontemplation) and Proactive maintenance. In the present study the original dimensions gave higher Cronbach’s alphas than the Braathen dimensions. This was unexpected as the sample in our study should be considerably more similar to the sample in the study by Braathen et al. than the Canadian study, as both were done in Norway, and participants had similar diagnoses and sick leave duration. It should also be noted that the Cronbach`s alphas were not high for the three dimensions associated with work outcomes (between 0.59 and 0.65), indicating that the items making up these dimensions do not measure the same construct very well.

The associations with work outcomes were stronger for higher scores on the Prepared for action—self-evaluative dimension than the action—behavioral dimension. This was somewhat surprising as the Prepared for action—behavioral dimension is closer to RTW according to the Readiness for RTW model. A possible explanation is the wording of the items constituting the dimensions. The items in the Prepared for action—self-evaluative dimension can be viewed as more precise in describing a RTW plan than the items in the Prepared for action—behavioral dimension, as it includes items like “you have a date for your first day back at work” and “you are not ready to go back to work” (reversely scored). The Prepared for action—behavioral items on the other hand are less precise. For example: “you are actively doing things to get back to work” and “you are getting help from others to return to work”. This could explain the low Cronbach’s alpha (0.59) for the Prepared for action—behavioral dimension, which indicates that this dimension was not well captured by the items used, at least in this sample.

The stage allocation method, categorizing participants to the dimension where they reported the highest score, was problematic in this sample as several participants tied between different dimensions. Franche et al. [7] solved this by placing participants with ties in the least advanced dimension towards RTW. In our study, however, 13% of the participants in the not working group had their highest score on two dimensions that were not adjacent. Most of these were between Contemplation and Prepared for action—behavioral. The items included in the Contemplation dimension are quite generic: “I have been wondering if there is something I could do to return to work”, “I wish I had more ideas about how to get back to work”, and “I would like to have some advice about how to get back to work”. These are questions most people on long term sick leave would ask themselves, regardless of where they are in their RTW process. This was supported by the fact that about 50% of the participants scored 4 or higher on this item both at the start and the end of the program, with no statistically significant change. Hence, we suggest that the items in the Contemplation dimension should be revised. After excluding participants with ties between dimensions, participants were poorly distributed across the stages. This is in line with the previous studies; Braathen et al. [8] had to exclude two stages from the analyses due to low number of participants and Franche et al. [7] excluded one stage. In addition, for individuals working, there was a higher probability of sustainable RTW for those allocated to the Uncertain maintenance stage than the Proactive maintenance stage, which is contradictory to the Readiness for RTW model.

The problems related to the stage allocation approach in this study might indicate that the Readiness for RTW scale in its present form does not satisfactory capture the stages of the RTW process. Another possibility is that the RTW process cannot be based on the same theories that describe other health behavior changes like smoking cessation. Obviously, motivational factors do play a role in any behavioral change, also when deciding to RTW after sick leave. However, the major difference between the RTW process and other health behavior changes may be the importance of contextual factors. Workplace factors like work demands, supervisor support and possibilities for work modifications and temporary flexible part-time work may be just as important for the RTW decision, as the readiness of the employee [15, 20–24].

In line with previous studies for both musculoskeletal complaints [15] and common mental health disorders [16, 25], the single question assessing the participants’ expectations about length of sick leave was associated with work outcomes. Models including the single expectation question were better than models including the Readiness for RTW dimensions for work outcomes. Therefore, if the goal is to just predict RTW, our results indicate that the single expectation question should be preferred over the Readiness for RTW questionnaire. However, the Readiness for RTW scale was also developed to assess the individual’s stage of readiness for RTW and not merely predict RTW. Still, the results of the present study suggest that more research is needed before it can be considered for clinical use.

Another application of the Readiness for RTW scale proposed by Franche et al. [7] is evaluation of RTW interventions. In this study we found that three of the dimensions changed during the interventions: Prepared for action—self-evaluative, Prepared for action—behavioral and Proactive maintenance, but their change scores were not associated with work outcomes. This could partly be due to lack of statistical power, as those who only filled out one of the questionnaires or changed category (and therefore filled out part B of the questionnaire) could not be included in the analysis. The lack of association could also be due to lack of effect of the rehabilitation programs, and that there seems to be a floor and ceiling problem for some of the dimensions. The observed changes in scores were small, and there are currently no established values for clinically significant changes.

### Strengths and Limitations

Due to the low number of participants achieving sustainable RTW we were not able to calculate areas under the receiver operating characteristic (ROC) curve, which would have been useful when comparing the dimensions in the Readiness for RTW scale with the single expectation question. Another limitation in this study, and a problem with the Readiness for RTW scale, is the possible misclassification of participants. In Norway, graded sick leave is commonly used and the question “are you currently back at work”, that determined if the participants received the “not working” or “working” questionnaire could be misunderstood in regard to whether it means working at all or working as normal. Also, 21% of the participants in the not working group did not have a job to return to and might relate differently to the questions than people who have a job. We did not have enough participants to do a subgroup analysis for this group. Another limitation was the number of missing questionnaires, 24% at the start of the rehabilitation programs and 35% at the end of the programs. However, we do not expect that the non-responders differ in the association between the Readiness for RTW scale and RTW compared to the responders. Hence, the missing questionnaires should not significantly affect the results besides the loss of statistical power. A third methodological consideration was the use of the dimensions as continuous variables. Some of them did not entirely meet the linearity assumption. The way we chose to categorize the variables gave a low number of participants in some categories, and therefore some categories had to be excluded from the analyses. Furthermore, using categorized variables also restricted the possibility to adjust for possible confounders. Therefore, we chose to report the dimensions as continuous variables in the main analyses, which mean that estimations should be interpreted with caution. A major strength of this study was the use of registry data on sick leave, which ensured no missing data or recall bias.

## Conclusion

Three dimensions of the Readiness for RTW scale were associated with RTW outcomes; Prepared for action—self-evaluative, Prepared for action—behavioral and Uncertain maintenance, and could be useful screening tools in determining appropriate RTW measures. However, several weaknesses with the Readiness for RTW scale were established; high scores on the most advanced dimension towards RTW was not the one that predicted work outcomes best and stage allocation was problematic due to several ties between not necessarily adjacent dimensions in the RTW-process. Models including the Readiness for RTW dimensions were generally not as good at explaining work outcomes as models including a single expectation question. Therefore, more research and probably revision of the instrument is needed if the Readiness for RTW scale is to be used for evaluation of interventions and as a useful tool in clinical settings.

## Electronic supplementary material

Below is the link to the electronic supplementary material.

Supplementary material 1 (DOCX 15 KB)

Supplementary material 2 (DOCX 14 KB)

Supplementary material 3 (DOCX 16 KB)

Supplementary material 4 (DOCX 13 KB)

Supplementary material 5 (DOCX 16 KB)
