# Supplementary material for: Associations Between the Readiness for Return to Work Scale and Return to Work: A Prospective Study
Source: J Occup Rehabil. 2017 Mar 16;28(1):97–106. doi: 10.1007/s10926-017-9705-2 (PMC5820391; doi:10.1007/s10926-017-9705-2)
Supplement: Supplementary file 1 — Supplementary material 1 (DOCX 15 KB) [file 10926_2017_9705_MOESM1_ESM.docx]

**Online resource 1** The Readiness for Return to Work questionnaire

| **Not working sample** |
| --- |
| Precontemplation dimension items |
| I don`t think I will ever be able to go back to work (A1) |
| As far as I`m concerned, there is no point in thinking about returning to work (A4) |
| As far as I`m concerned, I don`t need to go back to work ever (A22) |
| Contemplation dimension items |
| I have been wondering if there is something I could do to return to work (A15) |
| I wish I had more ideas about how to get back to work (A20) |
| I would like to have some advice about how to get back to work (A21) |
| Prepared for action – self evaluative dimension items |
| Physically, I am starting to feel ready to go back to work (A9) |
| I am not ready to go back to work (R) (A12) |
| I have found strategies to make my work manageable so I can return to work (A13) |
| I have a date for my first day back at work (A18) |
| Prepared for action – behavioral dimension items |
| I am doing things actively now to get back to work (A6) |
| I have been increasing my activities at home in order to build up my strength to go back to work (A10) |
| I am getting help from others to return to work (A11) |
|  |
| **Working sample** |
| Uncertain maintenance dimension items |
| I am back at work but not sure I can keep up the effort (B8) |
| I worry about having to stop working again due to my health complaints* (B9) |
| I still find myself struggling to stay at work due to my health complaints* (B10) |
| I am back at work and it is going well (R) (B11) |
| I feel I may need help in order to stay at work (B12) |
| Proactive maintenance dimension items |
| I am doing everything I can to stay at work (B2) |
| I have learnt different ways to cope with my health complaints so that I can stay at work* (B5) |
| I am taking steps to prevent having to go off work due to my health complaints* (B6) |
| I have found strategies to make my work manageable so I can stay at work (B7) |
|  |

R: Item scale reversed

* “health complaints” was “injury” or “pain” in the original questionnaire.
