# Supplementary material for: Associations Between the Readiness for Return to Work Scale and Return to Work: A Prospective Study
Source: J Occup Rehabil. 2017 Mar 16;28(1):97–106. doi: 10.1007/s10926-017-9705-2 (PMC5820391; doi:10.1007/s10926-017-9705-2)
Supplement: Supplementary file 2 — Supplementary material 2 (DOCX 14 KB) [file 10926_2017_9705_MOESM2_ESM.docx]

**Online resource 2** Associations between the Readiness for RTW dimensions and work outcomes (sustainable RTW and work participation days) during 9 months follow-up as scores increase one unit.

|  | **Odds ratio for return to work (95% CI)^a^** | **Work participation days**  **(95% CI)^b^** |
| --- | --- | --- |
| **Not working** |  |  |
| Precontemplation (1-5) | 0.46 (0.18-1.15) | -17.78 (-38.22- 2.66) |
| Contemplation (1-5) | 0.91 (0.49- 1.68) | -10.83 (-28.30- 6.64) |
| Prepared for action- self evaluative (1-5) | 2.54 (1.52- 4.22) | 23.28 (11.92- 34.65) |
| Prepared for action-behavioral (1-5) | 2.11 (1.13- 3.95) | 20.71 (4.90- 36.51) |
|  |  |  |
| **Working** |  |  |
| Uncertain maintenance (1-5) | 0.33 (0.19- 0.58) | -18.17 (-26.49- -9.84) |
| Proactive maintenance (1-5) | 1.44 (0.72- 2.87) | 4.38 (-9.94- 18.70) |

For both samples N varied somewhat according to the number of missing information on each variable. Dimension scores measured at the end of rehabilitation.

^a^ Logistic regression adjusted for age, gender and education.

^b^ Linear regression adjusted for age, gender and education.
