# Supplementary material for: Associations Between the Readiness for Return to Work Scale and Return to Work: A Prospective Study
Source: J Occup Rehabil. 2017 Mar 16;28(1):97–106. doi: 10.1007/s10926-017-9705-2 (PMC5820391; doi:10.1007/s10926-017-9705-2)
Supplement: Supplementary file 3 — Supplementary material 3 (DOCX 16 KB) [file 10926_2017_9705_MOESM3_ESM.docx]

**Online resource 3** Associations between the Readiness for Return to Work stages (stage allocation) and work outcomes during 9 months of follow-up

|  | **Estimated work**  **participation days^a^**  (95% CI) | **Probability of**  **sustainable return to work^b^**  (95% CI) |
| --- | --- | --- |
| **Not working** | **n=71** | **n=71** |
| Precontemplation^c^ | - | - |
| Contemplation | 63.7 (44.2- 83.3) | 0.17 (0.06- 0.29) |
| Prepared for action- self evaluative^c^ | - | **-** |
| Prepared for action-behavioural | 100.9 (78.6-123.1) | 0.42 (0.25- 0.60) |
|  |  |  |
| **Working** | **n=116** | **n=116** |
| Uncertain maintenance | 151.3 (142.2-160.3) | 0.70 (0.61- 0.79) |
| Proactive maintenance | 133.5 (116.2- 150.8) | 0.45 (0.27- 0.64) |
|  |  |  |

Stage allocation based on scores on the Readiness for RTW scale at the end of rehabilitation.

^a^ Linear regression, adjustment for gender, age and education

^b^ Logistic regression, adjustment for gender, age and education

^c^ Excluded due to low number of participants
