# Supplementary material for: Associations Between the Readiness for Return to Work Scale and Return to Work: A Prospective Study
Source: J Occup Rehabil. 2017 Mar 16;28(1):97–106. doi: 10.1007/s10926-017-9705-2 (PMC5820391; doi:10.1007/s10926-017-9705-2)
Supplement: Supplementary file 4 — Supplementary material 4 (DOCX 13 KB) [file 10926_2017_9705_MOESM4_ESM.docx]

**Online resource 4** Scores on the Readiness for return to work dimensions at the beginning and at the end of rehabilitation.

|  | **Pre-scores (before the start of the program)**  median (IQR) | **Post-scores**  **(at the end of the program)**  median (IQR) | **Median change**  (IQR) | **Pre- vs post-scores p-value^a^** |
| --- | --- | --- | --- | --- |
| **Not working** |  |  |  |  |
| Precontemplation | 1.3 (1.0-1.7) | 1.3 (1.0-1.7) | 0 (-0.3- 0) | 0.333 |
| Contemplation | 4.0 (3.7-4.3) | 4.0 (3.7-4.3) | 0 (-0.3- 0.3) | 0.355 |
| Prepared for action- self evaluative | 2.5 (2.0-3.0) | 2.5 (2.0-3.5) | 0.3 (-0.3- 1) | 0.006 |
| Prepared for action-behavioral | 3.7 (3.3-4.0) | 4.0 (3.3-4.7) | 0.3 (-0.3- 0.7) | 0.014 |
| **Working** |  |  |  |  |
| Uncertain maintenance | 3.6 (3.0-4.0) | 3.4 (2.7- 4.0) | 0 (-0.4- 0.2) | 0.220 |
| Proactive maintenance | 4.0 (3.5-4.3) | 4.3 (4.0- 4.5) | 0.3 (0- 0.8) | <0.001 |

For both samples N varied somewhat according to the number of missing information on each variable.

^a^ Pre- and post-scores compared with Wilcoxon signed rank test
