# Supplementary material for: Associations Between the Readiness for Return to Work Scale and Return to Work: A Prospective Study
Source: J Occup Rehabil. 2017 Mar 16;28(1):97–106. doi: 10.1007/s10926-017-9705-2 (PMC5820391; doi:10.1007/s10926-017-9705-2)
Supplement: Supplementary file 5 — Supplementary material 5 (DOCX 16 KB) [file 10926_2017_9705_MOESM5_ESM.docx]

**Online resource 5** Measures of explained variance (adjusted R^2^ and pseudo R^2^) from the regression models for each of the outcomes used in the article - including separate models for each of the Readiness for RTW dimension scores, models with all the dimension scores included and models with the single expectation question.

|  | **Outcome measures** | |
| --- | --- | --- |
|  | **Work participation days^a^** | **Probability of sustainable RTW^b^** |
|  | Adjusted R^2^ | Pseudo R^2^ |
| **Not working** |  |  |
| Precontemplation | 0 | 0.02 |
| Contemplation | 0 | 0.01 |
| Prepared for action-  self-evaluative | 0.10 | 0.10 |
| Prepared for action-behavioral | 0.08 | 0.09 |
| All four dimensions^c^ | 0.13 | 0.13 |
| Expectations about  length of sick leave | 0.14 | 0.19 |
|  |  |  |
| **Working** |  |  |
| Uncertain maintenance | 0.17 | 0.18 |
| Proactive  maintenance | 0.03 | 0.05 |
| Both dimensions^d^ | 0.16 | 0.18 |
| Expectations about  length of sick leave | 0.24 | 0.23 |
|  |  |  |

^a^ Linear regression adjusted for age, gender and education.

^b^ Logistic regression adjusted for age, gender and education.

^c^ All four dimensions for participants not working included in the same model

^d^ Both dimensions for participants working included in the same model
